# The tricyclic antidepressant clomipramine inhibits neuronal autophagic flux

**DOI:** 10.1038/s41598-019-40887-x

**Published:** 2019-03-19

**Authors:** Federica Cavaliere, Alessandra Fornarelli, Fabio Bertan, Rossella Russo, Anaïs Marsal-Cots, Luigi Antonio Morrone, Annagrazia Adornetto, Maria Tiziana Corasaniti, Daniele Bano, Giacinto Bagetta, Pierluigi Nicotera

**Affiliations:** 1 German Center for Neurodegenerative Diseases (DZNE), Bonn, Germany; 2 Preclinical and Translational Pharmacology, Department of Pharmacy, Health and Nutritional Sciences, University of Calabria, Arcavacata di Rende (Cosenza), Italy; 3 Department of Health Sciences, University “Magna Graecia” of Catanzaro, Catanzaro, Italy

## Abstract

Antidepressants are commonly prescribed psychotropic substances for the symptomatic treatment of mood disorders. Their primary mechanism of action is the modulation of neurotransmission and the consequent accumulation of monoamines, such as serotonin and noradrenaline. However, antidepressants have additional molecular targets that, through multiple signaling cascades, may ultimately alter essential cellular processes. In this regard, it was previously demonstrated that clomipramine, a widely used FDA-approved tricyclic antidepressant, interferes with the autophagic flux and severely compromises the viability of tumorigenic cells upon cytotoxic stress. Consistent with this line of evidence, we report here that clomipramine undermines autophagosome formation and cargo degradation in primary dissociated neurons. A similar pattern was observed in the frontal cortex and liver of treated mice, as well as in the nematode *Caenorhabditis elegans* exposed to clomipramine. Together, our findings indicate that clomipramine may negatively regulate the autophagic flux in various tissues, with potential metabolic and functional implications for the homeostatic maintenance of differentiated cells.

## Introduction

Depression is a long-term, disabling condition affecting more than 350 million people worldwide^1^. The number of diagnosed individuals with mood disorders is constantly increasing each year. Apart from psychiatric syndromes, depressive states are commonly manifested in patients affected by neurodegenerative diseases^2^. As a consequence, antidepressants are widely prescribed drugs across an array of neurological disorders^3^. Antidepressants are a heterogeneous group of compounds, which can be divided into four distinct categories, depending on their primary mechanism of action: norepinephrine re-uptake inhibitors (NRIs), selective serotonin re-uptake inhibitors (SSRIs), serotonin/norepinephrine re-uptake inhibitors (SNRIs) and monoamine oxidase inhibitors (MAOIs). A fifth group comprises atypical antidepressants, such as the unicyclic aminoketone bupropion (i.e., norepinephrine-dopamine re-uptake inhibitor) and the noradrenergic and specific serotonergic antidepressant mirtazapine^4^. Among the first antidepressant drugs launched on the market, the tricyclic antidepressants (TCAs) act primarily as SNRIs^5^.

As noted above, the primary action of most antidepressants involves the increase of monoamine concentration in the neuronal synaptic space^4^. While the modulation of monoamine concentration is quite rapid, the therapeutic response takes several weeks. This line of evidence has suggested that other molecular processes may contribute to the retarded therapeutic outcome of the antidepressants^6–8^. In support of this hypothesis, antidepressants have been demonstrated to possess a large spectrum of biological properties^4,6,9,10^.

Autophagy is an evolutionarily conserved homeostatic process that crucially regulates cellular function and maintenance^11^. Activation of the autophagic pathway results in the degradation of long-lived proteins and organelles^12^. This process is constitutively active at basal levels and can be further induced by a variety of stimuli, including environmental and cellular stressors. Notably, it has been suggested that autophagic stimulation can diminish the formation and accumulation of intracellular protein aggregates or insoluble inclusions^13–16^. The loss of intracellular proteostasis is particularly deleterious in the nervous system and has been associated with many forms of neurodegenerative disorders, including Alzheimer’s disease, Parkinson’s disease and Huntington’s disease^17,18^. The importance of autophagy to neuronal maintenance has been further highlighted by evidence in transgenic mice, in which genetic suppression of the autophagy-related proteins ATG-5 or ATG-7 compromises the autophagic pathway, negatively affects cellular viability, causes neuronal degeneration and leads to premature death^19,20^. It was previously reported that exposure of tumorigenic cell lines to tricyclic antidepressant clomipramine inhibits the degradation of the autophagic cargo^21,22^. It remains unclear whether clomipramine may also affect autophagy in postmitotic cells. In the present study, we provide evidence that clomipramine blocks the autophagic flux in primary neuronal culture. Consistently, we show that clomipramine negatively alters autophagy *in vivo* in three-weeks treated mice as well as in nematodes. Taken together, long-term treatment with tricyclic antidepressants may influence autophagy, and therefore cellular homeostasis, in the central nervous system. Further investigations and evaluations are warranted to determine the possible pathophysiological implications in common idiopathic neurodegenerative diseases.

## Materials and Methods

### Animal procedures and *in vivo* mouse treatment

All animal work was approved and performed in conformity to the guidelines of the State Agency for Nature, Environment and Consumer Protection in North Rhine Westphalia (LANUV) and of the Italian Ministry of Health for Animal care (DM 116/1992). In all our experiments, we used C57BL/6 J mice that were purchased from Charles River Laboratories (Germany and Italy), housed under a 12 h light–dark cycle and allowed *ad libitum* access to food and water. Mice were used at 6 weeks of age and 22 to 25 g of weight. Mice were treated intraperitoneally with clomipramine hydrochloride (20 mg/kg) or fluoxetine hyrochloride (10 and 30 mg/kg) for 21 days and according to previous published protocols^23,24^. For *in vivo* experiments, we used 7 males per group. Control mice were injected with an equivalent volume of saline solution. All adult animals included in this study were sacrificed by cervical dislocation and, when required, embryos were removed by caesarean section.

### LC3 and p62 formation in *ex vivo*

Brain tissues were incubated in Dulbecco’s Modified Eagle’s Medium (DMEM), supplemented with 10% FCS and incubated at 37 °C, with 5% CO_2_. To block lysosomal proteases, tissues were exposed to NH_4_Cl (20 mM, Sigma-Aldrich) and leupeptin (200 μM, Sigma-Aldrich)^25^. After 2 h incubation, tissues were separately collected from each well and centrifuged at 1000 g for 5 min at 4 °C. The obtained pellet was homogenized in an appropriate volume of 0.25 M sucrose (supplemented with protease inhibitors), sonicated and processed for protein quantification.

### Cell Cultures

Primary cortical neurons were prepared from E17.5 pregnant mice as described previously^26^. Dissociated neurons were plated on 100 μg/ml poly-L-lysine (MW > 300 kDa) coated dishes at a density of about 4•10^5^ cells/ ml (12-well plate), cultured at 37 °C and at 5% CO_2_. After 2 h, the medium was completely removed and neurons were maintained in Neurobasal Medium supplemented with 2% B27, 2 mM L-glutamine, 100 U/l penicillin and 100 μg/ml streptomycin. Cytosine arabinoside (10 μM) was added at 5 days *in vitro* in order to inhibit the cells mitotic division. Cortical neurons were routinely used between day 6 and 8.

### Chemicals and cultures treatment

Both clomipramine and fluoxetine (Sigma-Aldrich) were prepared in 100% DMSO at 10 mM final concentration and diluted in PBS immediately before use. Where indicated, PBS-diluted clomipramine, fluoxetine (1 and 5 µM, final concentration) and/or bafilomycin A1 (Baf A1, 20 nM; Sigma-Aldrich) were added to the cellular medium. Control cells were treated with the corresponding volume of vehicle (i.e., DMSO + PBS).

### Immunoblot analysis

Neuronal cells and tissue samples were directly lysed in boiling Laemmli buffer (60 mM Tris-HCl, pH 6.8; 2% SDS; 10% glycerol; 5% beta-mercaptoethanol; 0.01% bromophenol blue). Nematodes were collected and resuspended in RIPA lysis buffer (Sigma-Aldrich). After sonication, equal amount of total proteins was separated by SDS polyacrylamide gel electrophoresis (12% to 15% percentage of acrylamide in the running gel) and transferred onto nitrocellulose membranes. Membranes were incubated for 1 h at room temperature (RT) with 5% non-fat milk in Tris-buffered saline, containing 0.05% Tween-20. Primary and secondary HRP-conjugated antibodies were incubated in the same buffer. Protein specific signals were detected using ECL Western Pico Detection system (ThermoFisher Scientific) and chemiluminescence signal visualized using Chemidoc imaging system (Biorad). The following primary antibodies and dilutions were used: guinea-pig polyclonal anti-p62 (1:1000; Progen), rabbit polyclonal anti-LC3B (1:1500; Sigma-Aldrich), mouse monoclonal anti-β-actin (1:5000; Sigma-Aldrich) and mouse monoclonal anti-actin (1:5000, Abcam). HRP-conjugated goat anti-mouse, anti-rabbit and anti guinea pig IgG (ThermoFisher Scientific, Waltham) were used as secondary antibodies.

### *Caenorhabditis elegans* strains and methods

The following strains were used: wild type N2, AM141 *rmIs133 [unc-54p::Q40::YFP]*, CL2120 *dvIs 14 [(pCL12) unc-54::beta 1–42* + *(pCL26) mtl-2::GFP]*, DA2123 *adIs2122[lgg-1p::GFP::lgg-1+rol-6(su1006)]*. Nematode Growth Medium (NGM) plates were seeded with *E*. *coli* strain OP50 as a food source and kept at 20 °C. NGM agar plates containing clomipramine and fluoxetine (final concentration of 10 or 50 μg/ml) were kept at 4 °C and used within one week. Drug concentrations were chosen based on previous protocols^27–30^. Synchronized L3 larvae or young adult nematodes were transferred to drug-containing agar plates for 1 to 7 days as indicated in the text.

### Statistical analysis

Data were expressed as the mean ± S.E.M of the indicated experiment numbers. Statistical analysis was evaluated by ANOVA followed by Dunnett’s test for multiple comparisons. Where indicated, Student’s t test was used to evaluate differences between two means.

## Results

### Clomipramine and fluoxetine reduce autophagy in neuronal primary cultures

**Figure 1:**
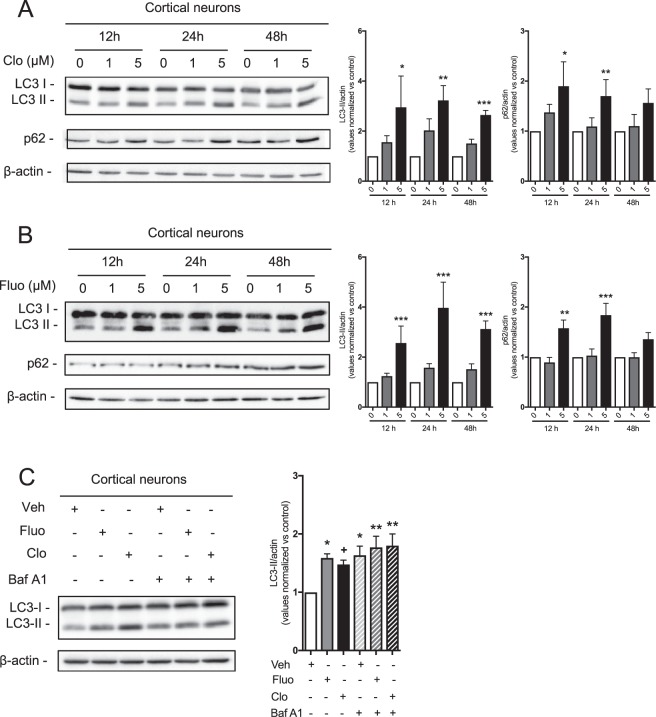
Clomipramine and fluoxetine treatments reduce autophagy flux in cortical neurons. **(A**,**B**) Primary cortical neurons were treated with (**A**) clomipramine or (**B**) fluoxetine at concentrations of 1 and 5 μM for 12, 24 and 48 h. Densitometric analysis of LC3-II (left) and p62 (right) is reported. β-Actin was used as loading control. Bars represent mean ± S.E.M. Each group results from 6 independent neuronal cultures. (**C**) Cortical neurons were incubated with clomipramine (5 μM) or fluoxetine (5 μM) for 12 h, while they were exposed to 20 nM Baf A1 for only 3 h. Densitometric analysis of LC3-II represents mean ± S.E.M of 4 independent neuronal cultures (^**+**^*p* = 0.0867, *p < 0.05, **p < 0.01, ***p < 0.001).

Autophagy requires the formation and the expansion of phagophores^31^. An important step in the autophagosome maturation is the conjugation of microtubule-associated protein 1 light chain 3 (LC3-I, 18 kDa) to phosphatidylethanolamine. The conversion of LC3-I to the lipidated membrane-bound LC3-II (16k Da) is used as a marker for autophagosome formation^32,33^. Following a previous line of evidence in tumorigenic cells^21,22^, we set off to determine whether clomipramine alters neuronal autophagy. We initially used primary cortical neurons exposed to increasing concentrations (1 and 5 μM) of clomipramine. We also used fluoxetine, a SSRI with a chemical structure completely unrelated to tricyclics, since we aimed to define whether the effect was specific for clomipramine or a general property of antidepressants. We found that both compounds enhanced the LC3-I conversion to LC3-II in a concentration-dependent manner at all analyzed time points (Fig. 1A,B). Higher levels of LC3-II normally indicate an increased autophagosome number^33^. However, this can be ascribed to an increased formation or a decreased degradation of LC3-II containing vesicles. To discriminate between these two possibilities, we assessed the degradation of the protein cargo p62, which is a substrate that accumulates in autophagy deficient cells^34,35^. Similar to the enhanced LC3-II conversion, we found that p62 levels increased following treatment with both antidepressants in a concentration-dependent manner (Fig. 1A,B). To further support our line of evidence, we exposed primary neuronal cultures to the V-ATPase inhibitor bafilomycin A1 (Baf A1)^36^. Baf A1 blocks lysosomal acidification and prevents the fusion between autophagosomes and lysosomes^37^, leading to a higher amount of LC3-II when autophagy flux is accelerated in the presence of autophagy activators^38^. While 20 nM Baf A1 treatment induced a significant increase of LC3-II conversion, co-treatment of cortical neurons with clomipramine or fluoxetine and Baf A1 did not enhance further LC3-II accumulation (Fig. 1C). This evidence strongly suggests that clomipramine and fluoxetine inhibit the autophagic flux in primary dissociated neurons rather than increase the autophagic rate. Taken together, these data suggest that clomipramine and fluoxetine negatively regulate neuronal autophagic pathway in primary cultured cells.

### Clomipramine decreases autophagic flux in murine tissues

To assess whether antidepressant treatment affects autophagy *in vivo* in mammals, chronic intraperitoneal (i.p.) administration of clomipramine and fluoxetine was performed in mice for 21 days. We assessed LC3-II and p62 levels in the presence or absence of lysosomal inhibitors using an assay previously adopted in similar experimental settings^39–41^. Lysosomal inhibitors act on lysosomal proteases, blocking their activity, thus preventing cargo degradation. Consequently, incubation with such inhibitors is informative of the autophagosome degradation rate.

**Figure 2:**
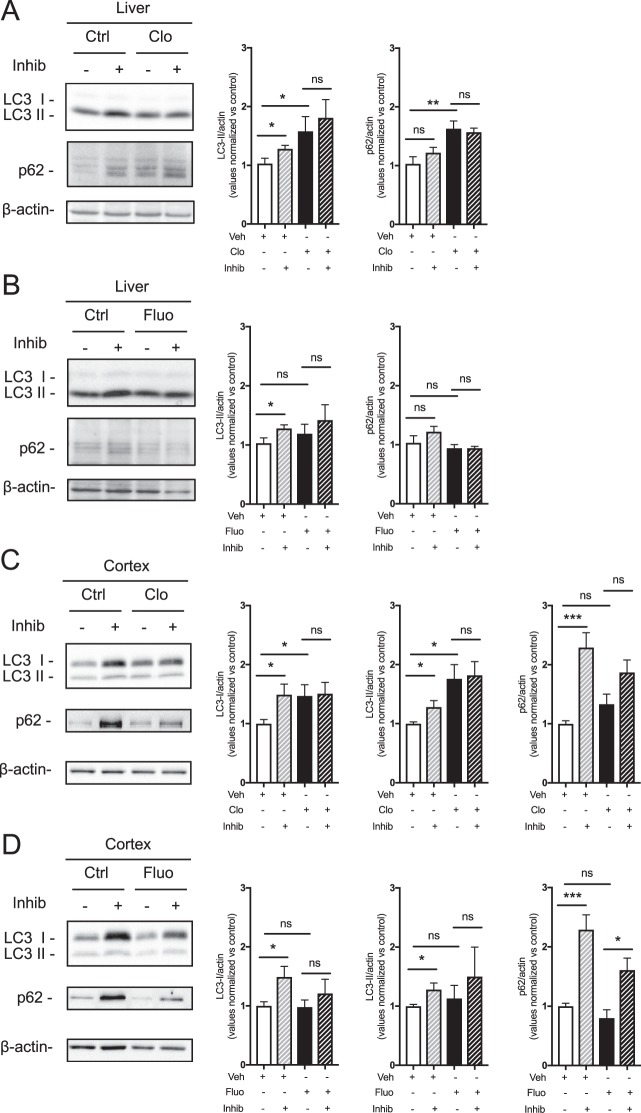
Autophagic flux is decreased by clomipramine treatment in mouse tissues. LC3-II and p62 levels in the **(A**,**B**) liver and (**C**,**D**) frontal cortex of mice treated with (**A**–**C**) clomipramine (Clo) or (**B**–**D**) fluoxetine (Fluo), compared to untreated animals (Ctrl). Data are relative to *ex vivo* tissues incubated with (+) and without (−) NH_4_Cl and leupeptin (inhib). β-Actin was used as a loading control. For the densitometric analysis, bars represent mean ± S.E.M. of 7 mice for each group (*p < 0.05, **p < 0.01, ***p < 0.001).

We initially tested the autophagic flux rate *ex vivo* in liver. Since liver is the organ mainly responsible for clomipramine^42^ and fluoxetine metabolism^43^, we reasoned that this tissue would have been definitely influenced by the two antidepressants. At the basal level, a very little amount of LC3-I levels was detected in the liver, probably due to the high autophagic flux in this organ and the high conversion of LC3-I in LC3-II in the *ex vivo* assay. However, both LC3-II and p62 were significantly increased in the liver of clomipramine treated mice compared to vehicle treated ones (Fig. 2A). Following incubation with lysosomal protease inhibitors, a significant increase of LC3-II levels was detectable in the liver of vehicle treated mice indicating the presence of an active autophagic process (Fig. 2A). Conversely, neither LC3-II nor p62 accumulated in the samples from clomipramine treated mice incubated with lysosomal inhibitors as compared to the same samples in the absence of the inhibitors (Fig. 2A). These data suggest that blockade of the autophagic flux was already occurring in the liver of the animals treated with the tricyclic antidepressant. To our surprise and against our previous findings in cortical neurons, fluoxetine treatment did not increase LC3-II and p62 levels in the liver of treated mice (Fig. 2B). These data demonstrate that impairment of the autophagic pathway does not occur in the liver of fluoxetine-treated animals.

Next, we assessed the autophagic flux in the frontal cortex of antidepressant treated mice as compared to vehicle treated. In line with what observed in liver, clomipramine treatment led to an increased LC3-II and p62 protein levels in murine frontal cortex (Fig. 2C). As expected, *in ex vivo* incubation with lysosomal inhibitors was associated with a significant accumulation of LC3-II and p62 compared to vehicle treated mice, while little effect was observed in the brain samples from clomipramine-treated mice (Fig. 2C). On the contrary, fluoxetine treatment did not alter the basal levels of LC3-II and p62 (Fig. 2D). In the samples from fluoxetine-treated mice and following incubation with the lysosomal inhibitors, the significant accumulation of p62 indicated an active autophagic flux (Fig. 2D). The same effects were observed with a higher dose of fluoxetine (i.e., 30 mg/kg i.p., data not shown), suggesting that the absence of effects with this SSRI was not dose-related. Overall, our findings suggest that clomipramine, but not fluoxetine, impairs autophagy in the brain when chronically administered to mice.

### Clomipramine treatment increases intracellular aggregates in *C*. *elegans*

**Figure 3:**
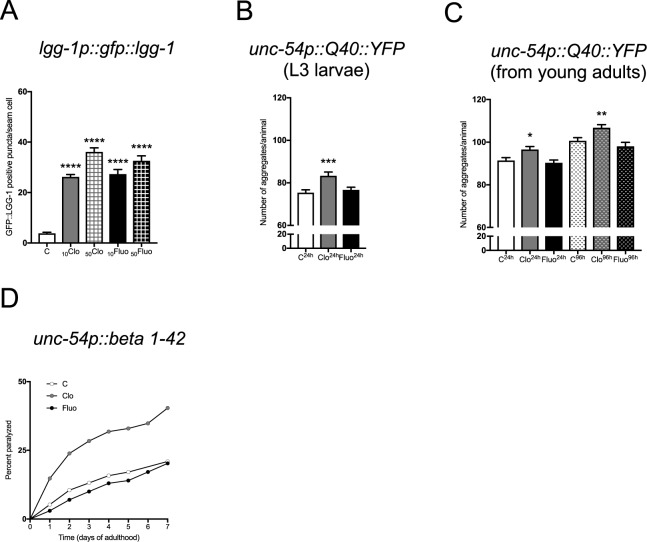
Clomipramine and fluoxetine modulate *C*. *elegans* autophagy in a different manner. (**A**) Quantification of GFP::LGG-1 positive puncta at the confocal microscope (n animals = 60, ****p < 0.0001). Nematodes overexpressing *lgg-1p::gfp::lgg-1* were exposed to clomipramine and fluoxetine for 24 h. Data information = _10_Clo and _10_Fluo = 10 μg/ml in NGM agar; _50_Clo and _50_Fluo = 50 μg/ml in NGM agar; C = control (equivalent volume of DMSO as vehicle). (**B**,**C**) Quantification of Q40::YFP containing puncta in nematodes upon treatment with 10 μg/ml clomipramine or fluoxetine. (**B**) L3 larvae expressing *unc-54p::Q40::YFP* transgene were exposed to antidepressants for (**B**) 24 h (n animals = 40, ***p < 0.0001), while (**C**) young adults were grown for 24 h and 96 h on drug-containing NGM agar plates (n animals = 40, *p < 0.05 **p < 0.01). (**D**) Percentage of paralyzed nematodes overexpressing human β-amyloid peptide. Animals were treated for 7 consecutive days with 10 μg/ml clomipramine or fluoxetine (n animals = 150, **p < 0.01). Percentage of paralyzed animals was determined every 24 h.

To determine the evolutionarily conserved mechanism of clomipramine and fluoxetine, we performed a series of experiments in the nematode *C*. *elegans*. We initially assessed autophagosome formation in *C*. *elegans* carrying the transgene *lgg-1p::GFP::lgg-1*^44^ and exposed to two concentrations (i.e., 10 and 50 μg/ml) of clomipramine and fluoxetine for 24 h. LGG-1 is the mammalian ortholog of LC3 and is recruited to nascent autophagosomes. In our experimental conditions, 24 h incubation of L3 larvae with clomipramine and fluoxetine resulted in an increased number of GFP::LGG-1 positive puncta, suggesting that both clomipramine and fluoxetine affected autophagosome formation in nematodes (Fig. 3A and Table 1). We reasoned that block of autophagy would promote the accumulation of insoluble intracellular species in long-lived cells^17,45,46^, including in *C*. *elegans* tissues^47^. Thus, we assessed the cytotoxic consequence of clomipramine and fluoxetine treatment in animals expressing aggregate-prone proteins. To do so, we used nematodes overexpressing a YFP tagged to polyglutamine expansions (i.e., Q40::YFP) in the body wall muscle cells^48–50^. Over time, these animals display motility defects due to the accumulation of protein aggregates. We found that clomipramine treatment resulted in a time-dependent increased formation of polyQ-YFP-positive puncta (Fig. 3B,C and Table 1). On the contrary, fluoxetine treatment did not affect the formation of polyQ-YFP-positive puncta compared to untreated nematodes (Fig. 3B,C and Table 1). To support these findings with an alternative model of proteotoxicity, we employed a *C*. *elegans* strain overexpressing human β-amyloid peptide that causes cytotoxicity in muscle cells^51^. Consistent with the data above, we found that clomipramine significantly increased the percentage of paralyzed animals, while fluoxetine had almost an opposite effect, since it partially ameliorated mobility compared to untreated nematodes (Fig. 3D and Table 1). Taken together, our findings suggest that clomipramine treatment impairs autophagy and, consequently, affects proteostasis in *C*. *elegans*. Conversely, fluoxetine may stimulate autophagy in nematodes, resulting in an efficient maintenance of the proteome during aging.

**Table 1:** Statistic of Figure 3.

Fig.	Test	Factor	F(DFn,Dfd)	p-value	Sum	Post hoc correction	Mean 1	Mean 2	*p* value	Sum
3A	One-way ANOVA	Treatment	19.74 (4, 259)	<0.0001	****	C vs. _10_Clo C vs. _50_Clo C vs._10_Fluo C vs._50_Fluo	3.839 3.839 3.839 3.839	26.22 36.12 27.36 32.61	<0.0001 <0.0001 <0.0001 <0.0001	**** **** **** ****
3B	One-way ANOVA	Treatment	2.164 (2, 114)	0.0003	***	C^24h^ vs. Clo^24h^ C^24h^ vs. Fluo^24h^	75.38 75.38	83.38 76.74	0.0003 0.7297	*** ns
3C	Two-way ANOVA	Interaction Time Treatment	0.3581 (2, 233) 57.38 (1, 233) 13.88 (2, 233)	0.6994 <0.0001 <0.0001	ns **** ****	C^24h^ vs. Clo^24h^ C^24h^ vs. Fluo^24h^ C^96h^ vs. Clo^96h^ C^96h^ vs. Fluo^96h^	91.42 91.42 100.6 100.6	96.57 90.31 106.8 98.08	0.0274 0.8246 0.0070 0.3953	* ns ** ns
3D	Survival Curves	Clomipramine Fluoxetine		0.0023 0.8604	** ns					

## Discussion

Clomipramine is a tricyclic antidepressant that influences serotonergic neurotransmission. Although clomipramine has been used for short- and long-term treatment of many mental illness, it has shown to be particularly effective for obsessive-compulsive disorders. Several reports suggest that antidepressant drugs interfere with the autophagic process^21,22,52–54^, however all previous studies were conducted in dividing cells. Here, we focused our work on two antidepressants with unrelated chemical structures. We provide evidence that both clomipramine and fluoxetine can inhibit autophagy *in vitro* in primary dissociated neurons. However, only chronic clomipramine treatment can affect the autophagic flux in frontal cortex and liver as revealed by a well-established *ex vivo* assay^39,40,55^. To support further this set of observations, we extended our work to nematodes. We found that, in an evolutionarily conserved manner, clomipramine stimulates the accumulation of GFP::LGG-1-positive autophagosomes. Moreover, clomipramine increases the accumulation of polyQ-containing and Aβ intracellular inclusions, suggesting an impairment in the global proteostasis and consequent formation of insoluble proteinaceous deposits. Antidepressant clomipramine may inhibit autophagy because of its basic and lipophilic properties. As such, we would expect that other antidepressants may have a similar effect. Indeed, being lipophilic amines, some antidepressants accumulate into acidic compartment (e.g., lysosomes)^56–58^ as it has been reported in tumorigenic cells treated with several of these compounds^59^. This enrichment in lysosomes may affect vesicular pH and block cargo degradation^37,60–62^ as observed for the antimalarial chloroquine^60,63^. Based on this line of evidence, one explanation of our findings is that the inhibition of the autophagic flux is potentially due to altered lysosomal acidification. We cannot rule out that additional mechanisms, other than lysosomotropism, may participate in the modulation of autophagy, since clomipramine seems to impair autophagy in mouse tissues as well as in nematodes, while fluoxetine does not. Equally relevant, the absence of substantial autophagic defects in fluoxetine-treated animals may be due to the different pharmacokinetic (i.e., metabolism and volume of distribution) of fluoxetine compared to clomipramine. Although further studies are warranted to dissect the molecular mechanisms underlying clomipramine-mediated autophagic inhibition, we cautiously envisage that our findings may have some implications. For example, since the duration of antidepressant treatments can last for decades in some patients, several tissues would suffer of the burden of altered autophagic flux, potentially predisposing organs to proteotoxicity and consequent damage. This scenario fits with the knowledge that impairment of autophagy results in inefficient protein clearance^15,40,46,64^ and, as a consequence, may predispose to idiopathic neurodegenerative diseases^17,45,65^. Moreover, since autophagy is a process that is progressively reduced during aging^15,66,67^, certain antidepressants (e.g., clomipramine) may have an adverse effect to human healthspan^68^, especially to elderly individuals. Preclinical studies in rodents indicate that clomipramine negatively affects hippocampus-dependent spatial learning and memory^69^, however it remains uncertain their effects in humans due to to the lack of conclusive epidemiologic evidence. In this scenario, the consequence of long-term clomipramine treatment would be even more relevant in patients already affected by neurodegenerative disorders, as the high incidence of depression and agitation symptoms in patients suffering of Alzheimer’s or Parkinson’s disease often leads to the chronic use of antidepressants^70,71^. It is reasonable to assume that a further impairment of the cellular proteostasis may be detrimental and contribute to the progression of the pathology in these subjects^72–74^. In support of this hypothesis, it seems that some, but not all, psychotropic medications may induce a more rapid cognitive decline in people affected by Alzheimer’s or Parkinson’s diseases^75,76^. This issue remains a long-standing debate because other studies indicate positive or no effects of these drugs on cognition^77,78^.

In summary, our findings demonstrate that clomipramine treatment reduces neuronal autophagic flux in primary dissociated neurons. Moreover, chronic treatment with clomipramine causes autophagy deficiency in the liver and brain of mice. In a consistent manner, clomipramine enhances the number of autophagosomes and inhibits the degradation of aggregate-prone proteins in *C*. *elegans*. We recognize the importance of antidepressants in the treatment of psychiatric syndromes, as well as the repurposing of some of these existing compounds for fast-track development of novel therapeutic alternatives. While we acknowledge the limitations of our findings mainly based on experimental models, their underlying molecular meanings merit attention. Although the pathophysiological consequences of long-term clomipramine treatment require further studies in preclinical models and, more importantly, in humans, our study confirms further that certain tricyclic antidepressants (i.e., clomipramine) may be negative regulators of homeostatic processes that are critical for neuronal maintenance and function, with potential implications for certain forms of brain disorders and in subjects at risk of neurodegenerative diseases.

## Supplementary information

Supplementary Information
