# Supplementary material for: The tricyclic antidepressant clomipramine inhibits neuronal autophagic flux
Source: Sci Rep. 2019 Mar 19;9:4881. doi: 10.1038/s41598-019-40887-x (PMC6424961; doi:10.1038/s41598-019-40887-x)

Title: The tricyclic antidepressant clomipramine inhibits neuronal autophagic flux
Author list: Federica Cavaliere, Alessandra Fornarelli, Fabio Bertan, Rossella Russo, Anaïs Marsal-Cots, Luigi Antonio Morrone, Annagrazia Adornetto, Maria Tiziana Corasaniti, Daniele Bano, Giacinto Bagetta, and Pierluigi Nicotera

Supplemental Figure S1: uncropped immunoblots, Figure 1

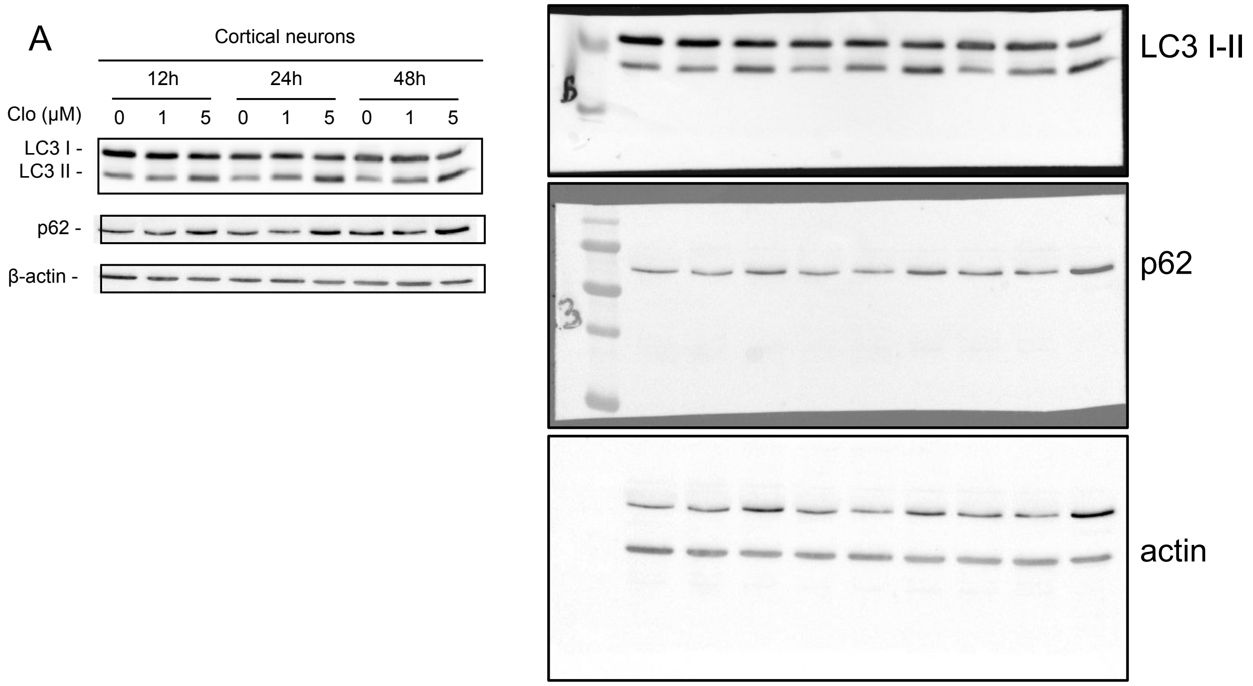

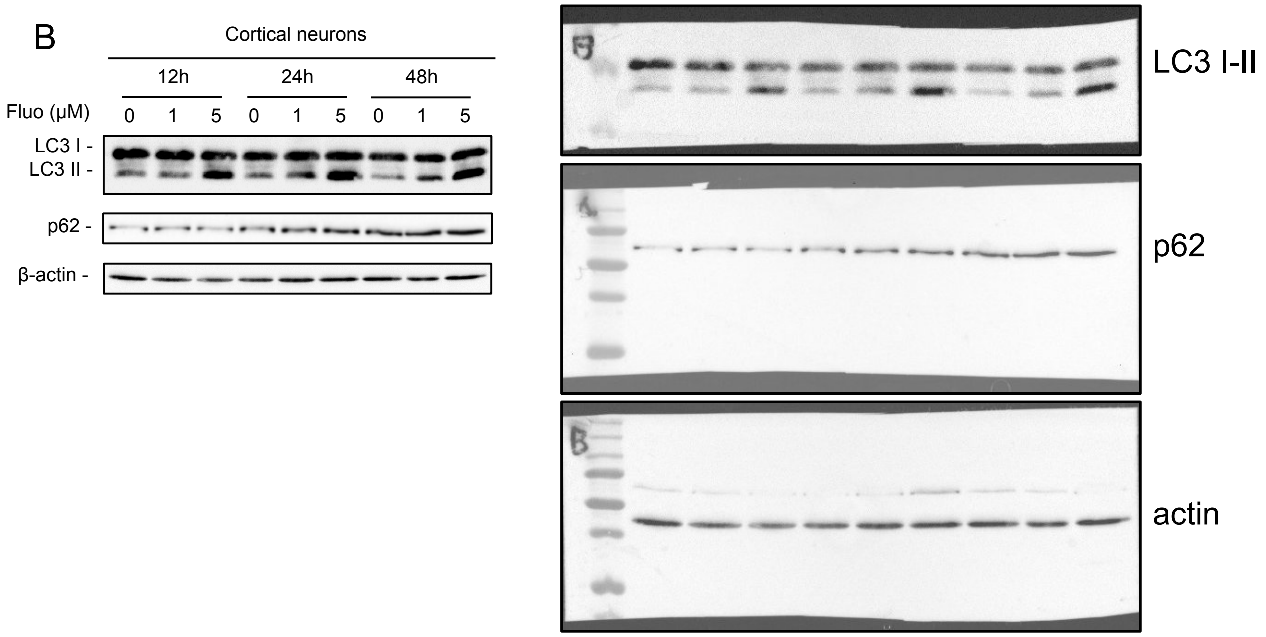

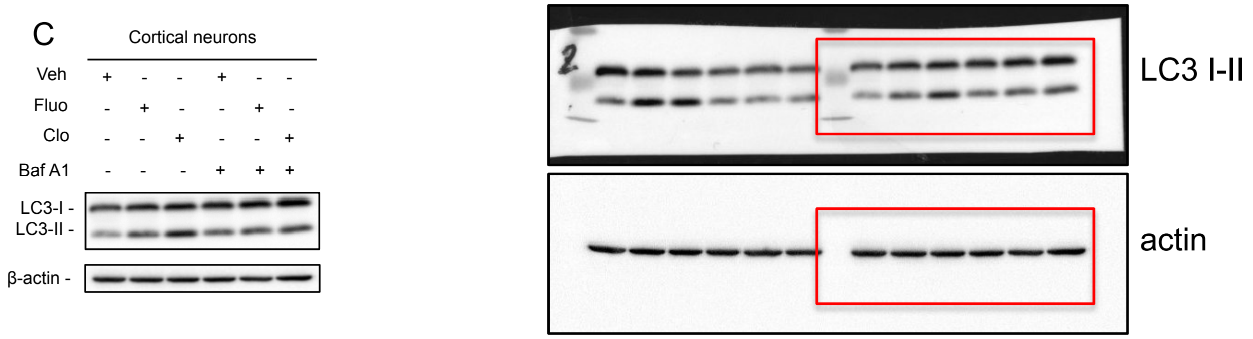

Title: The tricyclic antidepressant clomipramine inhibits neuronal autophagic flux
Author list: Federica Cavaliere, Alessandra Fornarelli, Fabio Bertan, Rossella Russo, Anaïs Marsal-Cots, Luigi Antonio Morrone, Annagrazia Adornetto, Maria Tiziana Corasaniti, Daniele Bano, Giacinto Bagetta, and Pierluigi Nicotera

Supplemental Figure S2: uncropped immunoblots, Figure 2

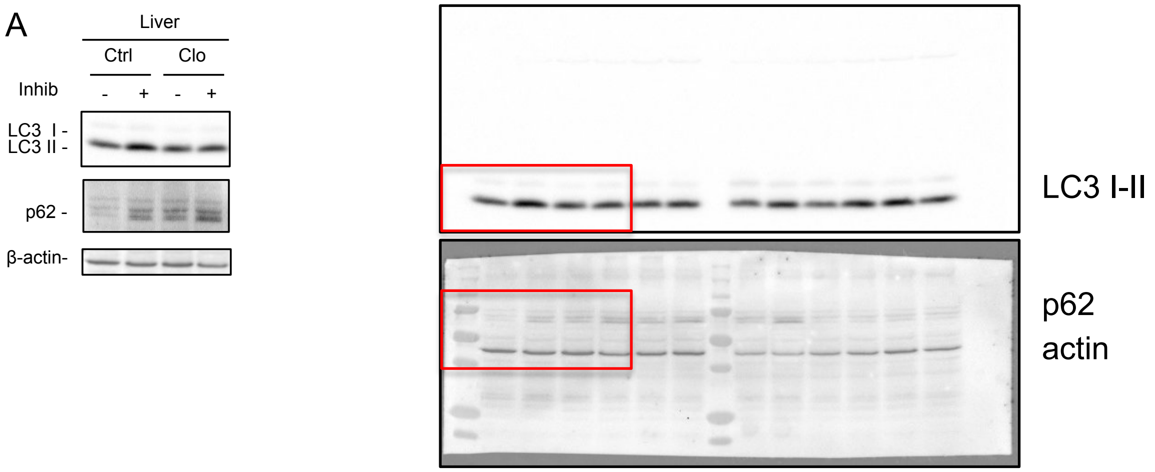

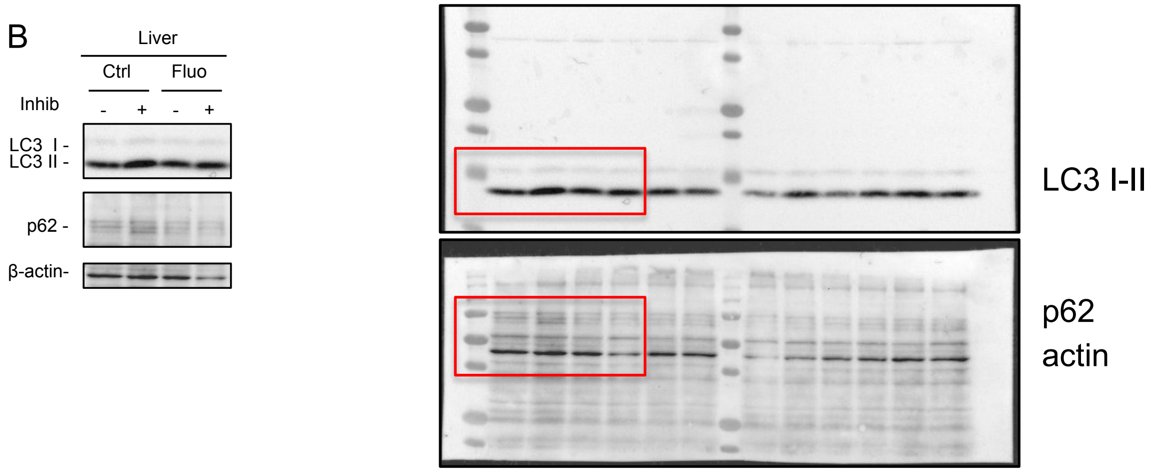

Title: The tricyclic antidepressant clomipramine inhibits neuronal autophagic flux
Author list: Federica Cavaliere, Alessandra Fornarelli, Fabio Bertan, Rossella Russo, Anaïs Marsal-Cots, Luigi Antonio Morrone, Annagrazia Adornetto, Maria Tiziana Corasaniti, Daniele Bano, Giacinto Bagetta, and Pierluigi Nicotera

Supplemental Figure S3: uncropped immunoblots, Figure 2

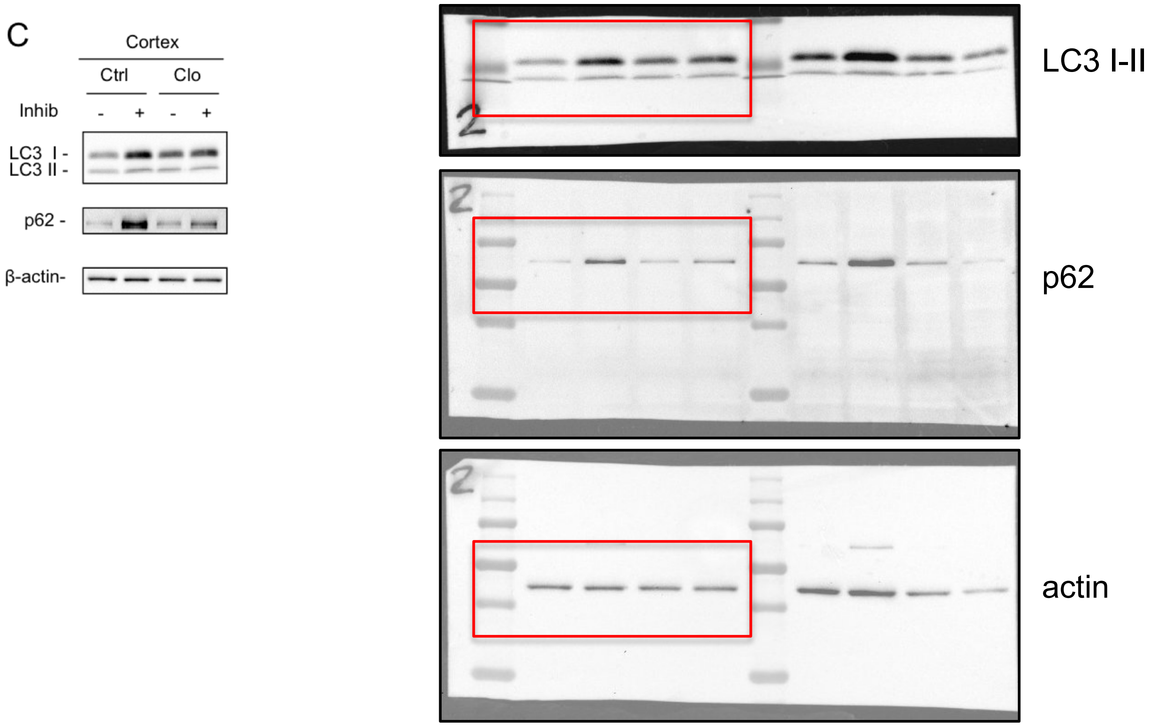

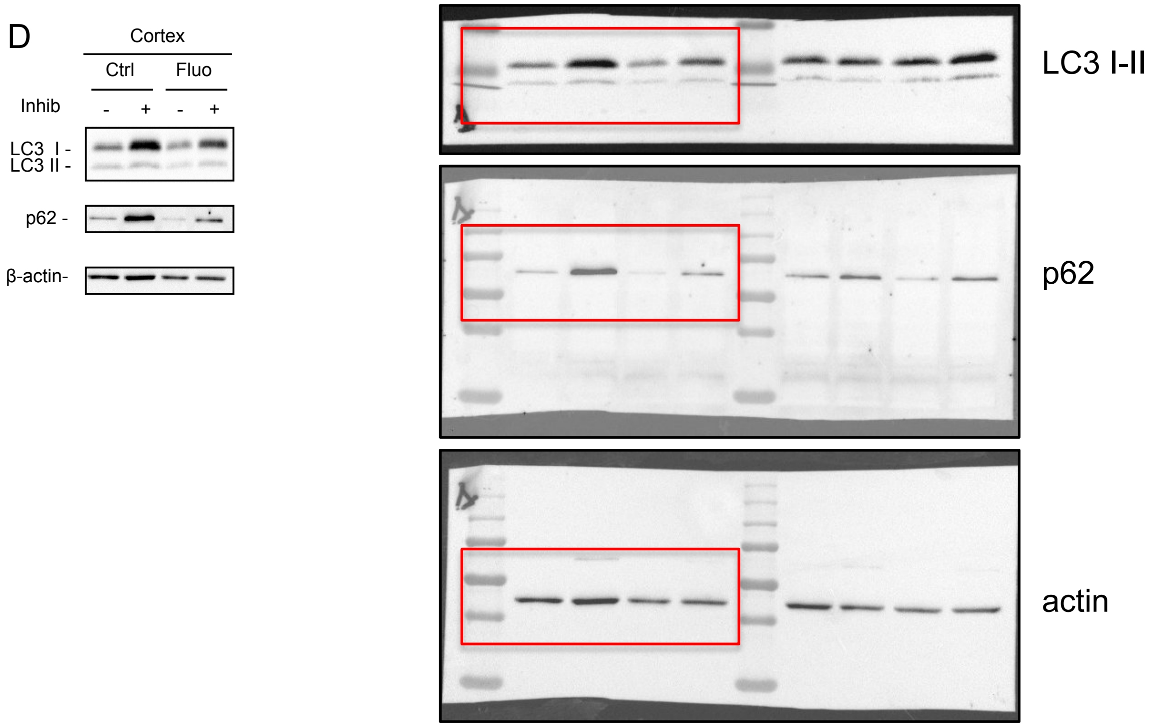

Supplement: Supplementary file 1 — Supplementary Information [file 41598_2019_40887_MOESM1_ESM.pdf]
